# Olanzapine and its use for methamphetamine-induced psychosis

**DOI:** 10.1192/j.eurpsy.2022.1853

**Published:** 2022-09-01

**Authors:** S. Nazir, A. Talpur, N. Hassan, U. Sharif

**Affiliations:** 1 Texas Tech University Health Sciences Center, Psychiatry, Lubbock, United States of America; 2 University of Louisville, Psychiatry, Louisville, United States of America; 3 Bronxcare Health System, Psychiatry, Newyork, United States of America; 4 Berkshire Medical Center, Psychiatry, Pittsfield, United States of America

**Keywords:** Psychosis, Methamphetamine, Methamphetamine induced psychosis, Olanzapine

## Abstract

**Introduction:**

Over time the prevalence of methamphetamine associated psychosis (MAP) has increased globally including Asia and Europe. Shoptaw et al looked at an RCT and concluded that olanzapine is superior to haloperidol in terms of tolerability and the side effect profile as it causes fewer extrapyramidal symptoms. Another study by Xue et al compared the efficacy of olanzapine and haloperidol and found that they had comparable effects but the onset time in the olanzapine group was significantly earlier than the haloperidol group. Srisurapanont et al analyzed 6 RCTs and concluded that quetiapine and olanzapine are probably superior than aripiprazole and risperidone.

**Objectives:**

The purpose of this review is to find out if olanzapine is better than other antipsychotics in treating methamphetamine-induced psychosis.

**Methods:**

PubMed, SCOPUS, and Web of Science literature databases were screened and filtered by using specific search terms, inclusion/exclusion criteria. Texts of the selected articles and trials were reviewed and the search terms generated a total of 248 results from the databases. After applying the criteria 200 citations were left and 15 papers were reviewed.

**Results:**

The literature review concluded that olanzapine can be used as an effective treatment for methamphetamine-induced psychosis. Olanzapine can help to reduce the psychotic symptoms in MAP with a quicker onset and lesser side effects.

**Conclusions:**

Olanzapine can help in the treatment of methamphetamine-associated psychosis and can be considered as the first-line therapy. Research is further needed with a higher pool of candidates in the future to compare the efficacy and tolerability of different typical and atypical antipsychotics.

**Disclosure:**

No significant relationships.

